# Sex-Specific Aspects of Skeletal Muscle Metabolism in the Clinical Context of Intensive Care Unit-Acquired Weakness

**DOI:** 10.3390/jcm11030846

**Published:** 2022-02-05

**Authors:** Lilian Jo Engelhardt, Julius J. Grunow, Tobias Wollersheim, Niklas M. Carbon, Felix Balzer, Joachim Spranger, Steffen Weber-Carstens

**Affiliations:** 1 Department of Anesthesiology and Operative Intensive Care Medicine (CCM/CVK), Charité–Universitätsmedizin Berlin, Corporate Member of Freie Universität Berlin, Humboldt-Universität zu Berlin, Augustenburger Platz 1, 13353 Berlin, Germany; lilian-jo.engelhardt@charite.de (L.J.E.); julius.grunow@charite.de (J.J.G.); tobias.wollersheim@charite.de (T.W.); niklas.carbon@charite.de (N.M.C.); 2 Institute of Medical Informatics, Charité–Universitätsmedizin Berlin, Corporate Member of Freie Universität Berlin, Humboldt-Universität zu Berlin, Charitéplatz 1, 10117 Berlin, Germany; felix.balzer@charite.de; 3 Department of Endocrinology and Metabolic Diseases, Charité–Universitätsmedizin Berlin, Corporate Member of Freie Universität Berlin, Humboldt-Universität zu Berlin, Charitéplatz 1, 10117 Berlin, Germany; joachim.spranger@charite.de

**Keywords:** intensive care unit-acquired weakness (ICUAW), critical illness myopathy (CIM), gender, sex, muscle, atrophy, metabolism, metabolites, insulin sensitivity, glucose

## Abstract

(1) Background: Female sex is considered a risk factor for Intensive Care Unit-Acquired Weakness (ICUAW). The aim is to investigate sex-specific aspects of skeletal muscle metabolism in the context of ICUAW. (2) Methods: This is a sex-specific sub-analysis from two prospectively conducted trials examining skeletal muscle metabolism and advanced muscle activating measures in critical illness. Muscle strength was assessed by Medical Research Council Score. The insulin sensitivity index was analyzed by hyperinsulinemic-euglycemic (HE) clamp. Muscular metabolites were studied by microdialysis. *M. vastus lateralis* biopsies were taken. The molecular analysis included protein degradation pathways. Morphology was assessed by myocyte cross-sectional area (MCSA). Multivariable linear regression models for the effect of sex on outcome parameters were performed. (3) Results: *n* = 83 (♂*n* = 57, 68.7%; ♀*n* = 26, 31.3%) ICU patients were included. ICUAW was present in 81.1%♂ and in 82.4%♀ at first awakening (*p* = 0.911) and in 59.5%♂ and in 70.6%♀ at ICU discharge (*p* = 0.432). Insulin sensitivity index was reduced more in women than in men (*p* = 0.026). Sex was significantly associated with insulin sensitivity index and MCSA of Type IIa fibers in the adjusted regression models. (4) Conclusion: This hypothesis-generating analysis suggests that more pronounced impairments in insulin sensitivity and lower MCSA of Type IIa fibers in critically ill women may be relevant for sex differences in ICUAW.

## 1. Introduction

Intensive Care Unit-Acquired Weakness (ICUAW) is a serious long-term consequence of critical illness associated with increased morbidity and mortality [1]. It is characterized by loss of muscle mass and functionality [2,3]. ICU-related physical impairments contribute to reduced quality of life and disabilities in survivors’ activities of daily living [4,5]. ICUAW is a risk factor for the post-intensive care syndrome (PICS), which is defined by physical and cognitive impairments as well as mental health problems [5]. Recently, attention has been drawn to PICS as a relevant burden in the COVID-19 pandemic [5].

Risk factors for ICUAW include severe sepsis with multiple organ failure, immobilization, use of neuromuscular blockers or corticosteroids as well as stress-induced hyperglycemia [6]. Furthermore, a multivariable analysis by De Jonghe et al. identified female sex as an independent predictor of ICU-acquired paresis [2]. The reasons for this remain unclear. Physiological differences in body composition, muscle strength, and energy metabolism may contribute [7].

Some pathomechanisms of ICUAW have already been investigated by our research group. Characteristic findings included Type II fiber atrophy, increase in atrophic genes, loss of myosin heavy chains, and inexitability of muscle membrane [3,6,8]. In addition, we found impairments in skeletal muscle glucose metabolism, such as reduced insulin sensitivity, disturbed GLUT4 translocation, and decreased stimulation of muscular glycolysis metabolites [9].

Skeletal muscle metabolism associated with ICUAW has not been studied from a sex-specific perspective. In this hypothesis-generating analysis, we will examine the pathomechanisms contributing to ICUAW in sex-specific groups, with a focus on skeletal muscle metabolism. To our knowledge, this is the first sex-specific study involving analysis of insulin sensitivity by hyperinsulinemic-euglycemic (HE) clamp, metabolites of muscular microdialysis, and molecular analysis of muscle biopsies in critically ill patients at high risk for ICUAW.

## 2. Materials and Methods

### 2.1. Study Design, Inclusion Criteria, and Setting

This is a sex-specific sub-analysis of two prospectively conducted observational and intervention ICU studies. First, the observational study examined skeletal muscle metabolism and Critical Illness Myopathy in the early phase of critical illness (ISRCTN77569430) [3,9]. Patients in the observational study received standard physiotherapy. This was followed by an intervention study to examine skeletal muscle metabolism and atrophy in ICU patients who received advanced early muscle-activating measures compared with protocol-based physiotherapy (ISRCTN19392591) [10,11]. The clinical investigations were approved by the Ethics Committee, Ethikkommission Charité. The setting was two ICUs of the tertiary care center Charité–Universitätsmedizin Berlin. Inclusion criteria were mechanically ventilated ICU patients ≥18 years with a SOFA Score ≥9 in the first 72 h after ICU admission. Exclusion criteria were patients with known diabetes mellitus, BMI > 35 kg/m^2^, known neuromuscular disorder, and infaust prognosis.

In our previous publications, the results were not reported in a sex-specific manner. This sub-analysis summarizes the results of both trials in groups of female and male ICU patients to identify differences in skeletal muscle metabolism during critical illness and to account for potential sex bias. In this sub-analysis, sex is defined according to the classification in the patient data management system. At the time point of data collection, this classification was binary. Non-binary gender identities were not assessed. The proportion of males and females and the measurements obtained per trial are shown in Figure S1.

The following measurements examined during the two trials were included in this sub-analysis.

### 2.2. Clinical Parameters

Routine clinical parameters from the local patient data management system: patient demographics (sex, age, BMI, weight, height, and severity of illness scores), diagnosis leading to ICU admission, mortality, medications, and nutrition. Muscle strength was assessed by Medical Research Council (MRC) Score at first awakening and at ICU discharge.

ICUAW was defined by median MRC Score <4. Frequency and time of physiotherapeutic sessions were investigated.

### 2.3. Glucose and Lipid Metabolism

#### 2.3.1. Insulin Sensitivity by Hyperinsulinemic-Euglycemic Clamp

Insulin sensitivity index (ISI) was determined by the gold standard technique of hyperinsulinemic-euglycemic (HE) clamp. Since endogen gluconeogenesis and glycogenolysis are suppressed by supraphysiological blood insulin concentrations during HE clamp, ISI is an indicator for peripheral insulin sensitivity. ISI is determined by the steady-state glucose infusion rate (mg/kg/min) divided by steady-state serum insulin concentration (mU/L) [12].

#### 2.3.2. Muscular Metabolites by Microdialysis in the *M. vastus lateralis*

Insulin-dependent stimulation of glucose metabolism was assessed by skeletal muscle microdialysis during HE clamp based on diffusion of metabolites across a semipermeable membrane. *CMA 600* Microdialysis AB (*Solna*, *Sweden*) was used. The Type 70 catheter was inserted into *M. vastus lateralis*. It contained an inlet tubing (perfusion fluid) connected to a pump and an outlet tubing (dialysate) leading to a microvial where the metabolites were collected. Metabolite analysis was based on enzymatic reactions. The metabolites glucose, pyruvate, lactate, and glycerol were measured in the *M. vastus lateralis* during HE clamp baseline and HE clamp steady-state.

### 2.4. Protein Degradation, Content, and Muscle Morphology

#### Molecular and Histological Analyses in Surgical Biopsies of *M. vastus lateralis*

Surgical muscle biopsies were obtained from *M. vastus lateralis* of ICU patients and healthy controls. Molecular and histological analysis has been described in detail previously [10]. The molecular analysis included real-time PCR and Western plot to determine mRNA expression of protein synthesis and degradation pathways and myosin heavy chains as well as myosin protein content. Muscle morphology was assessed by histological analysis of myocyte cross-sectional area (MCSA) analysis for Type I, IIa, and IIb fibers.

### 2.5. Statistical Analysis

Statistical analysis was performed using IBM Corp. Released 2020. IBM^®^ SPSS^®^ Statistics, Version 27. Armonk, NY. The Results were categorized into groups of the male and female sex. Results are reported as median with interquartile range or as absolute numbers with percentages. Non-parametric tests were used to compare variables between independent groups. The Mann–Whitney test was used for numerical variables and the Pearson–Chi2 for categorical variables. Multivariable linear regression models were conducted for the effect of sex on the outcome parameters insulin sensitivity, muscle atrophy gene expression (Calpain-1, Atrogin-1) and MCSA analysis for Type I, IIa, and IIb fibers. Regarding the categorical variable sex, male patients were defined as the reference in the multivariable linear regression analyses. The models were adjusted for the covariates BMI, age, and mean caloric intake per predicted body weight during ICU stay. *p*-values < 0.05 were considered statistically significant.

## 3. Results

Overall *n* = 83 critically ill patients were included in this pooled, sex-specific sub-analysis of the observational and interventional study. Fewer women were included in the studies, ♂*n* = 57, (68.7%) vs. ♀*n* = 26, (31.3%). Non-binary gender identities were not assessed. The sex distribution in groups in the observational and intervention study was:

- Observation: *n* = 33, ♂*n* = 24 (72.7%), ♀*n* = 9 (24.3%). Standard physiotherapy was performed.
- Intervention: *n* = 33, ♂*n* = 24 (72.7%), ♀*n* = 9 (24.3%). Advanced muscle-activating measures (e.g., electrical muscle stimulation, vibration therapy) were used in addition to protocol-based physiotherapy. The control arm of the interventional study included *n* = 17 patients, ♂*n* = 9 (52.9%), ♀*n* = 8 (47.1%), who received protocol-based physiotherapy. See Figure S1 for details.

The pooled results of the observational and intervention studies in groups of sex are presented in the following results.

### 3.1. Clinical Baseline Characteristics

Baseline data, caloric intake, and quantity of physiotherapy are shown in Table 1. Body height (*p* < 0.001) and weight (*p* = 0.001) were lower in female patients, while median BMI was 27 kg/m^2^ in both groups (*p* = 0.833). Mean caloric intake per predicted body weight was higher in females during ICU stay (*p* = 0.001) and in the first seven days after ICU admission (*p* = 0.008). Females received a higher insulin dose until the day of the muscle biopsy (*p* = 0.048). No sex differences were found in the frequency and duration of physiotherapy.

### 3.2. Clinical Diagnosis of ICUAW by MRC at First Awakening and ICU Discharge

MRC at first awakening and at ICU discharge was assessed in *n* = 37 male and *n* = 17 female patients. ICUAW by median MRC < 4 was present in *n* = 30 (81.1%) males and *n* = 14 (82.4%) females at first awakening (*p* = 0.911) and in *n* = 22 (59.5%) males and n = 12 (70.6%) females at ICU discharge (*p* = 0.432). No sex differences were observed in MRC at first awakening (♂3.3 (2.9/3.8) vs. ♀3.0 (2.4/3.6), *p* = 0.460) and at ICU discharge (♂ 3.9 (3.1/4.3) vs. ♀ 3.6 (3.3/4.0), *p* = 0.661). A separate sex-specific analysis of ICU by MRC in groups of observational and intervention trials identified no differences. Details are presented in Table S1.

### 3.3. Glucose and Lipid Metabolism

#### 3.3.1. Insulin Sensitivity Index (ISI) by Hyperinsulinemic-Euglycemic Clamp

**Figure 1:**
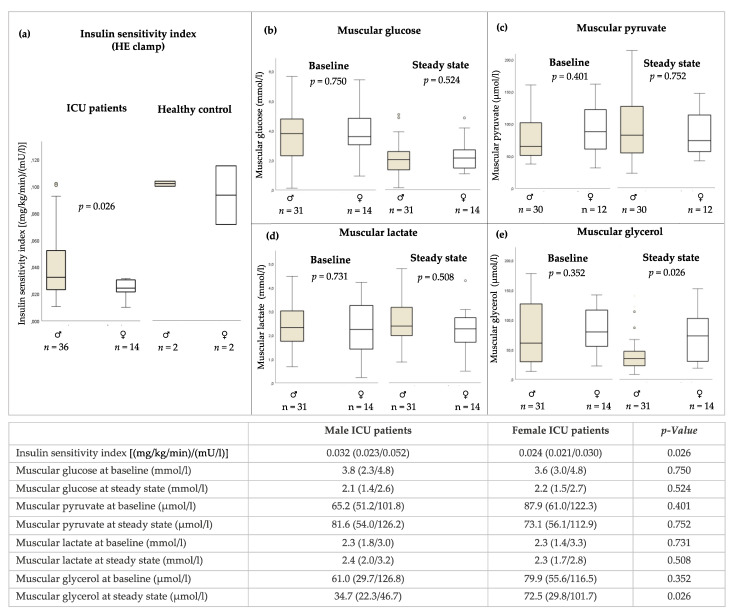
(**a**) Sex-specific insulin sensitivity index by HE clamp and metabolites of (**b**–**d**) glycolysis and (**e**) lipid metabolism by microdialysis in *M. vastus lateralis* during HE clamp. Missing measurements of metabolites occurred during microdialysis in *n* = 5 male patients. The number of patients for each measurement are given in the figure. The results are given as median with interquartile range. Mann–Whitney Test.

HE clamp was performed in *n* = 36 male and *n* = 14 female ICU patients (Figure 1a). The procedure was conducted on ICU Day 17 (15/21). Compared with healthy controls (♂*n* = 2, ♀*n* = 2), insulin sensitivity was impaired in male and female ICU patients. Insulin sensitivity at steady state of the HE clamp was reduced more in women, with a value of 0.024 (0.021/0.030) than in men, who had a value of 0.032 (0.023/0.052) (mg/kg/min)/(mU/L), *p* = 0.026). Steady-state plasma insulin levels were 174.7 (138.4/202.4) in males and 234.0 (191.8/265.5) in females (*p* = 0.010). Sex, age and mean caloric intake were all significantly associated with insulin sensitivity index in the multivariable model with the adjusted effect estimate for sex being β = −0.014 95% CI (−0.026 to −0.003). With R^2^ = 0.439 the model explained 43.9% of the overall variance of insulin sensitivity. Details are shown in Table 2.

#### 3.3.2. Muscular Metabolites of Glycolysis by Microdialysis in *M. vastus lateralis*

No sex differences were observed in muscular glucose levels and concentrations of metabolites of glycolysis (Figure 1b–d). In both groups, glucose decreased during the steady state of HE clamp compared to baseline (Figure 1b). The aerobe glycolysis metabolite pyruvate increased in male patients during the HE clamp, whereas it decreased in females (Figure 1c).

#### 3.3.3. Muscular Glycerol Concentrations by Microdialysis in *M. vastus lateralis*

The metabolite of fatty acid metabolism glycerol was significantly higher in female ICU patients during HE clamp at the steady state (*p* = 0.026) (Figure 1e).

Spearman’s correlation of circulating triglycerides and muscular glycerol by microdialysis in *M.vastus lateralis* as well as sex-specific analysis of circulating triglycerides is shown in Figure S2.

### 3.4. Protein Degradation, Content, and Muscle Morphology

#### 3.4.1. Molecular Analysis of Protein Degradation Pathways, Myosin Heavy Chains, and Myosin Protein Content in *M. vastus lateralis* Biopsies

**Figure 2:**
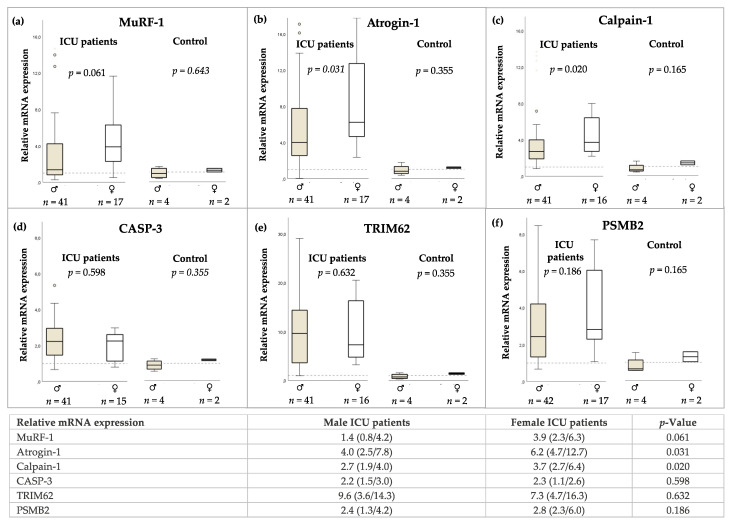
(**a**–**f**) Sex-specific relative mRNA expression of protein degradation atrogenes in biopsies of *M. vastus lateralis* in ICU patients and healthy control subjects. Results are given as median with interquartile range. Mann–Whitney Test.

Surgical muscle biopsies were extracted at median Day 15. The relative mRNA expression of protein degradation atrogenes compared to healthy controls is shown in Figure 2. Relative gene expression of the protein degradation atrogenes Atrogin-1 (*p* = 0.031) (Figure 2b) and Calpain-1 (*p* = 0.020) (Figure 2c) was higher in female ICU patients. Gene expression for atrogenes MuRF-1 (Figure 2a), CASP-3 (Figure 2d), TRIM62 (Figure 2e), and PSMB2 (Figure 2f) was probable in male and female patients. No differences were found in healthy control subjects. Sex was not significantly associated with muscle atrophy gene expression of Calpain-1 and Atrogin-1 in the unadjusted linear regression model and adjusted for age. Details are shown in Table S2.

**Figure 3:**
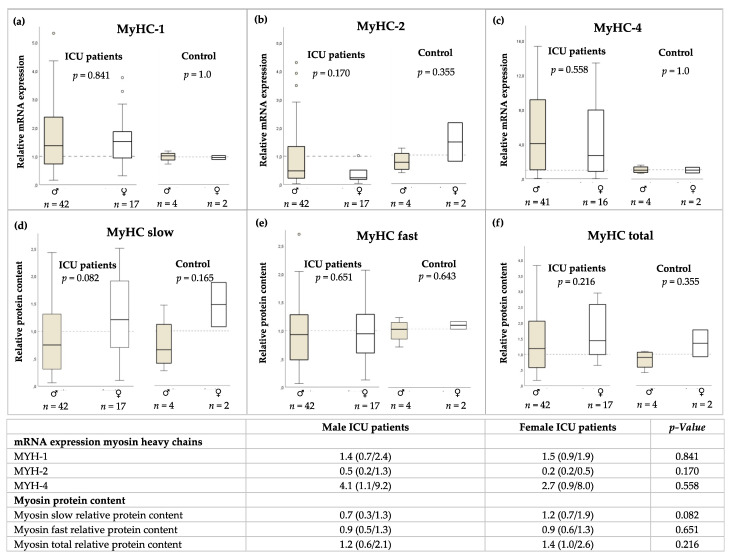
(**a**–**c**) Sex-specific relative mRNA expression of myosin heavy chains (MYH) and (**d**–**f**) relative myosin protein content in biopsies of *M. vastus lateralis* in ICU patients and healthy control subjects. Results are given as median with interquartile range. Mann–Whitney Test.

No sex differences were observed in the relative mRNA expression of myosin heavy chains (Figure 3a–c) and relative myosin protein content (Figure 3d–f) in ICU patients or healthy control patients.

#### 3.4.2. Histological Analysis of Surgical Biopsies of *M. vastus lateralis*—Myocyte Cross-Sectional Area (MCSA)

MCSA of all fiber types was significantly lower in female patients (Table 3). The differences were comparable to sex differences in MCSA of healthy persons published by Staron et al. [13]. Sex (β = −1345.6 95% CI (−2411.4 to −279.8)) and BMI (β = 161.7 95% CI (58.3 to 265.1)) were significantly associated with MCSA of Type IIa in the multivariable model. With R^2^ = 0.325 the model explained 32.5% of the overall variance of MCSA of Type IIa fibers. Sex was not significantly associated with MCSA of Type I and IIb fibers after adjusting for BMI, age, and caloric intake. Details are shown in Table 4.

## 4. Discussion

In this pooled sub-analysis, we examined sex differences in potential pathomechanisms of skeletal muscle metabolism in the clinical context of ICUAW. In this cohort of critically ill patients, sex-specific variations were observed in insulin sensitivity, muscular lipid metabolism, and muscle morphology. The adjusted regression model estimated a significant association of female sex with lower insulin sensitivity index and MCSA of Type IIa fibers.

### 4.1. Insulin Sensitivity

The insulin sensitivity index was most impaired in critically ill women. Sex was still associated with insulin sensitivity index when adjusted for age, caloric intake, and BMI in the multivariable linear regression analysis. With R^2^ = 0.439 the model explained 43.9% of the total variance in insulin sensitivity, roughly 30% more than in the unadjusted model. Previous data from our research group have shown that insulin sensitivity is impaired in ICU patients, which was particularly pronounced in patients with Critical Illness Myopathy [9]. This sex-specific analysis adds to the field that critically ill women were more severely affected by decreased insulin sensitivity. Severely impaired insulin sensitivity may be a contributing factor to the increased risk of ICUAW in women reported by De Jonghe [2]. The strength of our data is the measurements by the gold standard technique of the HE clamp, which is an elaborate procedure to perform in the ICU setting. In line with our finding, model-based calculations have shown lower insulin sensitivity in female ICU patients [14].

In particular, postmenopausal women, who make up the majority of women in the ICU due to their age, may be at higher risk for impaired insulin sensitivity. In healthy women, impaired insulin sensitivity has been noted after menopause, suggesting an influence of sex hormones [15,16,17]. Sharshar et al. investigated the effect of hormonal status on ICUAW and found a higher prevalence of hyperglycemia in postmenopausal females, which was associated with ICUAW [18]. This could be an indicator of impaired insulin sensitivity in older, critically ill women. However, there are no studies that have actually measured insulin sensitivity and assessed sex hormones in pre- and postmenopausal critically ill patients. Further studies are needed to investigate the effects of sex hormones on insulin sensitivity and muscle weakness during critical illness in fertile and postmenopausal patients. In clinical practice, elderly female patients could be an important target group for advanced muscle-activating measures to improve insulin sensitivity through exercise-induced glucose uptake. In addition, special attention should be paid to glycemic control in this cohort.

### 4.2. Muscular Substrate Metabolism

Impaired glucose metabolization in skeletal muscle cells could contribute to decreased insulin sensitivity in women. The aerobe glycolysis metabolite pyruvate in M. vastus lateralis increased only in men during HE clamp. Energetic failure at the enzymatic level due to decreased activity of glycolysis key enzymes may play a role. This has not been studied as a contributing factor in ICUAW, but it has been observed in nerve damage-induced muscle atrophy in rodents [19]. Interestingly, although neither pyruvate (aerobe glycolysis) nor lactate (anaerobe glycolysis) increased in the women, a decrease in muscular glucose was observed, suggesting insulin-dependent metabolization of glucose. Alternative pathways of glucose metabolization, such as glycogen synthesis, may have been stimulated in women.

Higher muscle glycerol concentrations by microdialysis during HE clamp suggest increased energy utilization of lipolysis substrates and decreased suppression of lipolysis by insulin in this cohort of critically ill women. Exogenous administration and endogenous release of catecholamines may contribute to an increase in glycerol utilization in critically ill women. Consistent with this, studies in healthy subjects have found increased glycerol turnover in women during infusion of ß- and alpha-adrenergic stimulation [20,21] and during exercise [22]. In addition, the muscular phenotype in females is described with a higher percentage of Type I muscle fibers utilizing primary mitochondrial oxidative phosphorylation, whereas males have more glycolytic fibers [23]. Circulating triglyceride concentrations measured during routine clinical practice did not correlate with glycerol measured in the skeletal muscle. However, interpretation is limited as triglycerides were not measured simultaneously to glycerol levels during microdialysis. 

Knowledge on sex-specific preferential muscle substrate metabolization may be relevant for diet composition to meet energetic requirements during critical illness. Concerning nutrition, sex-specific differences in resting energy expenditure must also be taken into account. Using indirect calorimetry, adipose (BMI ≥ 25 kg/m^2^), mechanically ventilated women were found to have significantly lower energy expenditure, adjusted for actual and ideal body weight, compared with men [24,25]. As per their BMI, the ICU patients in our cohort had a BMI of 27 kg/m^2^. However, men received a significantly lower caloric intake per predicted body weight, indicating a higher risk for undernutrition in critically ill male patients. In critically ill women, in addition to impaired insulin sensitivity, the higher caloric intake per predicted body weight in females may result in higher insulin requirements to achieve normoglycemia. Accordingly, in this cohort women received a higher insulin dose during the first two weeks of ICU stay (until muscle biopsy). In patients who do not receive indirect calorimetry, sex differences in energy expenditure, body height, and weight should be taken into account in the nutrition during critical illness to avoid a discrepancy between energy requirements and caloric intake.

### 4.3. Muscular Phenotype and Atrophy

Differences in muscular phenotype are discussed in a recent review of sex-specific muscle metabolism and atrophy by Rosa–Caldwell et al. [23]. Sex hormones may play a role in counteracting the upregulation of atrophy genes [23,26]. Interestingly, a prospective observational study of critically ill patients found an association between hypogonadism and ICUAW in men [18]. In our cohort, muscle atrophy gene expressions of Atrogin-1 and Calpain-1 were more severely upregulated in female patients only in the group comparison by non-parametric tests. A potential effect of sex on muscle atrophy gene expression was not observed in either the unadjusted regression model or when adjusted for age.

It is discussed that women are more prone to atrophy from disuse due to a higher proportion of Type I oxidative fibers [23]. Glycolytic fibers, which are found at a higher percentage in male muscle, tend to be more prone to inflammatory atrophy in cancer cachexia [23]. Our research group has found predominant atrophy of Type IIa myofibers in consecutive muscle biopsies in patients with ICUAW [3]. Interestingly, in this analysis a potential influence of sex on MCSA was exclusively observed for Type IIa fibers in the adjusted multivariable regression analysis. Females may be clinically more affected by ICUAW due to more pronounced atrophy of Type IIa myofibers and pre-existing lower MCSA of all fiber types [13].

Consecutive muscle biopsies in patients with risk for ICUAW may provide insight into whether the dynamics of fiber atrophy are more pronounced in females.

### 4.4. Limitations

This is a descriptive, hypothesis-generating analysis with single time point measurements. It is unclear whether the sex differences were pre-existing or due to critical illness. Although we excluded patients with diabetes mellitus, we cannot exclude the possibility of pre-existing impaired insulin sensitivity in women. Variable confounding factors may influence the observed sex differences. For example, age could potentially bias the observed sex differences for women, who tended to be slightly older. Though, sex seemed to independently impact on insulin sensitivity index and MCSA of Type IIa fibers after adjusting for age, BMI, and caloric intake. There are severe limitations due to the small size of the patient cohort. The multivariable regression analysis was narrowed to sex and three additional covariates to avoid overfitting of the model. In addition, more covariates would have resulted in missing values. Importantly, further studies with larger data sets, particularly of women, are, therefore, needed to conduct a more comprehensive and robust analysis, including multiple regression models, to draw consistent conclusions.

Because of the small number of patients per group, the observational and intervention studies were pooled. A possible sex-specific effect of advanced physiotherapeutic measures is therefore neglected in this sub-analysis. A separate sex analysis of the two studies was conducted by MRC for ICUAW during the review process. Sex differences in MRC scores were not found in either pooled or separate analyses of observational and intervention trials. This may be biased by the high incidence of ICUAW in this cohort of critically ill patients. While the underrepresentation of females compared to males in the observational study (♀27.3% vs.♂72.7%) and the intervention group (♀27.3% vs. ♂72.7%) of the intervention study was equal, the control group of the intervention study had a slightly higher proportion of women (♀52.9% vs.♂47.1%). This may have led to a sex bias when results of female and male patients were mixed in previous publications. The underrepresentation of critically ill women is most likely related to lower ICU admission rates among women [27].

## 5. Conclusions

In this cohort of non-diabetic ICU patients with a high risk for ICUAW, relevant sex differences in energy metabolism were found. This analysis suggests that more pronounced impairments in insulin sensitivity and lower MCSA of Type IIa muscle fibers in critically ill women may be relevant for sex differences in ICUAW. Due to the mentioned limitations, the data need to be interpreted with caution and clearly regarded as hypothesis-generating. It is important to consider gender/sex in future studies addressing metabolic and muscle-specific science issues in ICU patients.

## Figures and Tables

**Table 1:** Baseline characteristics.

	Male ICU Patients ♂*n* = 57 (68.7%)	Female ICU Patients ♀*n* = 26 (31.3%)	*p*-Value
Age (years)	49.0 (37.0/63.0)	60.5 (44.0/68.0)	0.082
BMI (kg/m^2^)	27.1 (23.4/29.8)	27.0 (23.1/31.2)	0.833
Height (cm)	178.0 (175.0/185.0)	165.0 (163.0/170.0)	<0.001
Weight (kg)	85.0 (80.0/96.0)	71.0 (65.0/85.0)	0.001
SOFA score at ICU admission	12.0 (10.0/14.0)	14.0 (11.0/16.0)	0.167
APACHE at ICU admission	21.0 (17.0/27.0)	23.5 (17.0/30.0)	0.170
SAPS 2 at ICU admission	52 (38.0/62.0)	54.5 (44.0/69.0)	0.107
Length of ICU stay (days)	29 (20.0/41.0)	27 (17.0/49.0)	0.933
Survival until ICU discharge (n, %)	45 (78.9)	24 (92.3)	0.132
**Reasons for ICU admission**			0.289
ARDS (n, %)	19 (33.3)	9 (34.6)	
Sepsis (n,%)	13 (22.8)	7 (26.9)	
Polytrauma (n,%)	16 (28.1)	3 (11.5)	
Neurological (n,%)	9 (15.8)	6 (23.1)	
Other (n,%)	-	1 (3.8)	
**Insulin dose and caloric intake**			
Insulin dose per body surface area during ICU stay (IE/m^2^)	18.5 (10.5/26.7)	20.1 (15.6/30.9)	0.289
Insulin dose per body surface until muscle biopsy (IE/m^2^)	18.6 (9.2/27.8)	30.0 (17.7/37.0)	0.048
Caloric intake per PBW during ICU stay (kcal/kgPBW)	18.5 (14.0/20.7)	21.6 (17.5/28.2)	0.001
Caloric intake per PBW ICU day 1–7 (kcal/kgPBW)	13.7 (10.5/19.1)	17.7 (13.5/24.9)	0.008
**Physiotherapy during ICU stay**			
Total duration of physiotherapy (min)	445 (267.5/662.5)	397.5 (265.0/740.0)	0.673
Time per physiotherapy session (min)	27.8 (24.8/29.9)	29.0 (24.0/30.3)	0.488
Number of physiotherapeutic sessions	16 (11/23.5)	17 (11/24)	0.701

Results are reported as median with interquartile range or as absolute numbers with percentages. BMI: Body Mass Index, SOFA: Sequential Organ Failure Assessment, ICU: Intensive Care Unit, ARDS: Acute Respiratory Distress Syndrome, PBW: Predicted Body Weight. Mann–Whitney Test and Pearson–Chi^2^ Test. Missing values: Time per physiotherapeutic session was not available for *n* = 2 males. No other missing values were found. Data on insulin dose per body surface area before muscle biopsy are reported in *n* = 42 male and *n* = 17 female patients who received biopsy.

**Table 2:** Multivariable linear regression for insulin sensitivity index.

Model 1 (Unadjusted)					Model 2				
Variable	*n*	B	95% CI B	*p*-Value	Variables	*n*	B	95% CI B	*p*-Value
Sex	50	−0.017	(−0.033 0.047)	0.014	Sex	50	−0.014	(−0.026 −0.003)	0.016
Constant		0.040	(−0.030 −0.003)	<0.001	BMI	50	−0.001	(−0.002 0.000)	0.231
					Age	50	−0.001	(−0.001 0.000)	<0.001
					Caloric intake	50	0.001	(0.000 0.002)	0.009
					Constant		0.067	(0.034 0.100)	<0.001
*n* = 50, R = 0.345, R^2^ = 0.119 (adjusted R^2^ = 0.101), *p* = 0.014					*n* = 50, R = 0.662, R^2^ = 0.439 (adjusted R^2^ = 0.389), *p* < 0.001				

**Table 3:** Myocyte cross-sectional area.

Myocyte Cross-Sectional Area (MCSA) *M. vastus lateralis*	Male ICU Patients ♂*n* = 37	Female ICU Patients ♀*n* = 17	*p*-Value
Mean MCSA Type I muscle fiber (µm^2^) Mean MCSA Type IIa muscle fiber (µm^2^) Mean MCSA Type IIb muscle fiber (µm^2^)	3844.4 (2999.7/4929.6)	2740.0 (2471.8/3591.1)	0.031
	4006.2 (2240.7/5002.2)	2036.3 (1675.4/3245.1)	0.003
	3229.7 (2080.6/4390.5)	2309.5 (1708.8/2958.8)	0.020
Results are given as median with interquartile range. MCSA myocyte cross-sectional area. Mann–Whitney Test.			
Myocyte cross-sectional area (MCSA) reference values from *M. vastus lateralis* in *n* = 95 healthy, untrained men, and *n* = 55 healthy, untrained women. Staron et al., Table 3. J Histochem Cytochem, 2000 [13]			
	**Healthy male** **♂*n* = 95**	**Healthy female** **♀*n* = 55**	
Mean MCSA Type I muscle fiber (µm^2^) Mean MCSA Type IIa muscle fiber (µm^2^) Mean MCSA Type IIb muscle fiber (µm^2^)	4844 ± 1286	4084 ± 895	
	6174 ± 1587 5160 ± 1324	3879 ± 867 3116 ± 792	
Values reported by Staron et al. are means ± standard deviation.			

**Table 4:** Multivariable linear regression model for myocyte cross-sectional area (MCSA).

Model 1 (Unadjusted)					Model 2				
**MCSA Type I Fibers**									
**Variable**	***n***	**B**	**95% CI B**	***p*-Value**	**Variables**	***n***	**B**	**95% CI B**	***p*-Value**
Sex	54	−904.4	(−1756.6 −52.3)	0.038	Sex	54	−793.1	(−1677.2 91.0)	0.078
Constant		4070.4	(3592.2 4548.5)	<0.001	BMI	54	103.4	(17.6 189.2)	0.019
					Age	54	1.0	(−24.0 26.0)	0.938
					Caloric intake	54	−37.2	(−97.2 22.8)	0.219
					Constant		1865.6	(−808.7 4539.8)	0.167
*n* = 54 ICU patients, R = 0.283, R^2^ = 0.08 (adjusted R^2^ = 0.063), *p* = 0.038					*n* = 54 ICU patients, R = 0.437, R^2^ = 0.191 (adjusted R^2^ =.125), *p* = 0.032				
**MCSA Type IIa Fibers**									
**Variable**	***n***	**B**	**95% CI B**	***p*** **-Value**	**Variables**	***n***	**B**	**95% CI B**	***p*** **-Value**
Sex	54	−1651.8	(−2730.9 −572.7)	0.003	Sex	54	−1345.6	(−2411.4 −279.8)	0.014
Constant		4027.2	(3421.8 4632.7)	<0.001	BMI	54	161.7	(58.3 265.1)	0.003
					Age	54	−20.2	(−50.4 9.9)	0.183
					Caloric intake	54	−49.3	(−121.7 23.0)	0.177
					Constant		1529.1	(−1694.7 4753.0)	0.345
*n* = 54 ICU patients, R = 0.392, R^2^ = 0.154 (adjusted R^2^ = 0.137), *p* = 0.003					*n* = 54 ICU patients, R = 0.570, R^2^ = 0.325 (adjusted R^2^ = 0.270), *p* < 0.001				
**MCSA Type IIb Fibers**									
**Variable**	***n***	**B**	**95% CI B**	***p*** **-Value**	**Variables**	***n***	**B**	**95% CI B**	***p*** **-Value**
Sex	54	−1161.7	(−2166.4 −157.1)	0.024	Sex	54	−960.1	(−1945.8 64.6)	0.067
Constant		3403.8	(2840.1 3967.5)	<0.001	BMI	54	123.1	(23.1 219.9)	0.017
					Age	54	−20.7	(−50.3 7.3)	0.159
					Caloric intake	54	−21.6	(−89.2 29.2)	0.538
					Constant		1485.7	(−1467.8 4733.3)	0.343
*n* = 54 ICU patients, R = 0.306, R^2^ = 0.094 (adjusted R^2^ = 0.076), *p* = 0.024					*n* = 54 ICU patients, R = 0.470, R^2^ = 0.221 (adjusted R^2^ = 0.158), *p* = 0.014				

## Data Availability

For ethical and data protection reasons, public disclosure of data is not possible. Editors, reviewers, and interested researchers can contact the corresponding author or sent a request to dai-researchdata@charite.de to get access to the data.

## Supplementary Materials

The following are available online at https://www.mdpi.com/2077-0383/11/3/846/s1, Figure S1: Sex distribution in the observational and intervention study.; Table S1: Sex-specific analysis of ICU by MRC in groups of observational and intervention trial.; Table S2: Multivariable linear regression model for atrophy gene expression of Calpain-1 and Atrogin-1.; Figure S2: (a) Spearman’s correlation of circulating triglycerides and muscular glycerol by microdialysis in M.vastus lateralis. (b) Sex-specific circulating triglycerides.

## References

[1] Van Aerde N., Meersseman P., Debaveye Y., Wilmer A., Gunst J., Casaer M.P., Bruyninckx F., Wouters P.J., Gosselink R., Van den Berghe G. (2020). Five-year impact of ICU-acquired neuromuscular complications: A prospective, observational study. Intensive Care Med..

[2] De Jonghe B., Sharshar T., Lefaucheur J.-P., Authier F.-J., Durand-Zaleski I., Boussarsar M., Cerf C., Renaud E., Mesrati F., Carlet J. Paresis Acquired in the Intensive Care Unit: A Prospective Multicenter Study. https://pubmed.ncbi.nlm.nih.gov/12472328/.

[3] Wollersheim T., Woehlecke J., Krebs M., Hamati J., Lodka D., Luther-Schroeder A., Langhans C., Haas K., Radtke T., Kleber C. (2014). Dynamics of myosin degradation in intensive care unit-acquired weakness during severe critical illness. Intensive Care Med..

[4] Thomas S., Mehrholz J. (2018). Health-related quality of life, participation, and physical and cognitive function of patients with intensive care unit-acquired muscle weakness 1 year after rehabilitation in Germany: The GymNAST cohort study. BMJ Open Br. Med. J. Publ. Group.

[5] Nakanishi N., Liu K., Kawakami D., Kawai Y., Morisawa T., Nishida T., Sumita H., Unoki T., Hifumi T., Iida Y. (2021). Post-Intensive Care Syndrome and Its New Challenges in Coronavirus Disease 2019 (COVID-19) Pandemic: A Review of Recent Advances and Perspectives. J. Clin. Med..

[6] Weber-Carstens S., Deja M., Koch S., Spranger J., Bubser F., Wernecke K.D., Spies C.D., Spuler S., Keh D. (2010). Risk factors in critical illness myopathy during the early course of critical illness: A prospective observational study. Crit. Care.

[7] Kautzky-Willer A., Harreiter J., Pacini G. (2016). Sex and Gender Differences in Risk, Pathophysiology and Complications of Type 2 Diabetes Mellitus. Endocr. Rev..

[8] Weber-Carstens S., Koch S., Spuler S., Spies C.D., Bubser F., Wernecke K.D., Deja M. (2009). Nonexcitable muscle membrane predicts intensive care unit-acquired paresis in mechanically ventilated, sedated patients. Crit. Care Med..

[9] Weber-Carstens S., Schneider J., Wollersheim T., Assmann A., Bierbrauer J., Marg A., Al Hasani H., Chadt A., Wenzel K., Koch S. (2013). Critical Illness Myopathy and GLUT4: Significance of Insulin and Muscle Contraction. Am. J. Respir. Crit. Care Med..

[10] Wollersheim T., Grunow J.J., Carbon N.M., Haas K., Malleike J., Ramme S.F., Schneider J., Spies C.D., Märdian S., Mai K. (2019). Muscle wasting and function after muscle activation and early protocol-based physiotherapy: An explorative trial. J. Cachexia Sarcopenia Muscle.

[11] Grunow J.J., Goll M., Carbon N.M., Liebl M.E., Weber-Carstens S., Wollersheim T. (2019). Differential contractile response of critically ill patients to neuromuscular electrical stimulation. Crit. Care.

[12] DeFronzo R.A., Tobin J.D., Andres R. (1979). Glucose clamp technique: A method for quantifying insulin secretion and resistance. Am. J. Physiol. -Endocrinol. Metab..

[13] Staron R.S., Hagerman F.C., Hikida R.S., Murray T.F., Hostler D.P., Crill M.T., Ragg K.E., Toma K. (2000). Fiber Type Composition of the Vastus Lateralis Muscle of Young Men and Women. J. Histochem. Cytochem..

[14] Uyttendaele V., Chase J.G., Knopp J.L., Gottlieb R., Shaw G.M., Desaive T. (2021). Insulin sensitivity in critically ill patients: Are women more insulin resistant?. Ann. Intensive Care.

[15] Yan H., Yang W., Zhou F., Li X., Pan Q., Shen Z., Han G., Newell-Fugate A., Tian Y., Majeti R. (2019). Estrogen Improves Insulin Sensitivity and Suppresses Gluconeogenesis via the Transcription Factor Foxo1. Diabetes.

[16] Manson J.E., Chlebowski R.T., Stefanick M.L., Aragaki A.K., Rossouw J.E., Prentice R.L., Anderson G., Howard B.V., Thomson C.A., LaCroix A.Z. (2013). Menopausal Hormone Therapy and Health Outcomes During the Intervention and Extended Poststopping Phases of the Women’s Health Initiative Randomized Trials. JAMA.

[17] Geer E.B., Shen W. (2009). Gender differences in insulin resistance, body composition, and energy balance. Gend. Med..

[18] Sharshar T., Bastuji-Garin S., De Jonghe B., Stevens R.D., Polito A., Maxime V., Rodriguez P., Cerf C., Outin H., Touraine P. (2010). Hormonal status and ICU-acquired paresis in critically ill patients. Intensive Care Med..

[19] Langer H.T., Afzal S., Kempa S., Spuler S. (2020). Nerve damage induced skeletal muscle atrophy is associated with increased accumulation of intramuscular glucose and polyol pathway intermediates. Sci. Rep..

[20] Horton T.J., Dow S., Armstrong M., Donahoo W.T. (2009). Greater systemic lipolysis in women compared with men during moderate-dose infusion of epinephrine and/or norepinephrine. J. Appl. Physiol..

[21] Schmidt S.L., Bessesen D.H., Stotz S., Peelor F.F., Miller B.F., Horton T.J. (2014). Adrenergic control of lipolysis in women compared with men. J. Appl. Physiol..

[22] Horton T.J., Pagliassotti M.J., Hobbs K., Hill J.O. (1998). Fuel metabolism in men and women during and after long-duration exercise. J. Appl. Physiol..

[23] Rosa-Caldwell M.E., Greene N.P. (2019). Muscle metabolism and atrophy: Let’s talk about sex. Biol. Sex Differ. BioMed Cent..

[24] Drolz A., Wewalka M., Horvatits T., Fuhrmann V., Schneeweiss B., Trauner M., Zauner C. (2014). Gender-specific differences in energy metabolism during the initial phase of critical illness. Eur. J. Clin. Nutr. Nat. Publ. Group.

[25] Zauner A., Schneeweiss B., Kneidinger N., Lindner G., Zauner C. (2006). Weight-adjusted resting energy expenditure is not constant in critically ill patients. Intensive Care Med..

[26] Zhao W., Pan J., Zhao Z., Wu Y., Bauman W.A., Cardozo C.P. (2008). Testosterone protects against dexamethasone-induced muscle atrophy, protein degradation and MAFbx upregulation. J. Steroid Biochem. Mol. Biol..

[27] Hollinger A., Gayat E., Féliot E., Paugam-Burtz C., Fournier M.-C., Duranteau J., Lefrant J.-Y., Leone M., Jaber S., Mebazaa A. (2019). Gender and survival of critically ill patients: Results from the FROG-ICU study. Ann. Intensive Care.

