# Correction: Engelhardt et al. Sex-Specific Aspects of Skeletal Muscle Metabolism in the Clinical Context of Intensive Care Unit-Acquired Weakness. *J. Clin. Med*. 2022, *11*, 846

**DOI:** 10.3390/jcm11195496

**Published:** 2022-09-20

**Authors:** Lilian Jo Engelhardt, Julius J. Grunow, Tobias Wollersheim, Niklas M. Carbon, Felix Balzer, Joachim Spranger, Steffen Weber-Carstens

**Affiliations:** 1 Department of Anesthesiology and Operative Intensive Care Medicine (CCM/CVK), Charité–Universitätsmedizin Berlin, Corporate Member of Freie Universität Berlin, Humboldt-Universität zu Berlin, Augustenburger Platz 1, 13353 Berlin, Germany; 2 Institute of Medical Informatics, Charité–Universitätsmedizin Berlin, Corporate Member of Freie Universität Berlin, Humboldt-Universität zu Berlin, Charitéplatz 1, 10117 Berlin, Germany; 3 Department of Endocrinology and Metabolic Diseases, Charité–Universitätsmedizin Berlin, Corporate Member of Freie Universität Berlin, Humboldt-Universität zu Berlin, Charitéplatz 1, 10117 Berlin, Germany

## Text Correction

Due to an Editorial Office error during processing, a number of male and female symbols were incorrectly shown in the pdf version of the manuscript [1]. In particular, all male and female symbols were shown as male symbols.

A correction has been made to Abstract, Section 3: paragraph 1, Table 1 (cell in first line and third column), Section 3.2: paragraph 1, Section 3.3.1: paragraph 1, Table 3 (cell in first line and third column; cell in fourth line and third column), Section 4.4: paragraph 2.

The authors state that the scientific conclusions are unaffected. This correction was approved by the Academic Editor. The original publication has also been updated.
