# Supplementary material for: Sex-Specific Aspects of Skeletal Muscle Metabolism in the Clinical Context of Intensive Care Unit-Acquired Weakness
Source: J Clin Med. 2022 Feb 5;11(3):846. doi: 10.3390/jcm11030846 (PMC8836746; doi:10.3390/jcm11030846)
Supplement: Supplementary file 1 [file jcm-11-00846-s001.zip › jcm-1532369-supplementary.pdf]

Observational Trial	Intervention Trial	
**Observation** (n = 33)<br>♀ n = 9, ♂ n = 24<br><br>Physiotherapy: Standard	**Intervention** (n = 33)<br>♀ n = 9, ♂ n = 24<br><br>Physiotherapy: Protocol-based + advanced muscle activating measures	**Control** (n = 17)<br>♀ n = 8, ♂ n = 9<br><br>Physiotherapy: Protocol-based
**HE clamp and muscular microdialysis**<br>♀ n = 3, ♂ n = 19	**HE clamp and muscular microdialysis**<br>♀ n = 6, ♂ n = 14	**HE clamp and muscular microdialysis**<br>♀ n = 5, ♂ n = 3
**Muscle biopsy for molecular and histologic analysis**<br>♀ n = 5, ♂ n = 17	**Muscle biopsy for molecular and histologic analysis**<br>♀ n = 6, ♂ n = 20	**Muscle biopsy for molecular and histologic analysis**<br>♀ n = 6, ♂ n = 5
**MRC assessment for clinical ICUAW diagnosis**<br>♀ n = 5, ♂ n = 17	**MRC assessment for clinical ICUAW diagnosis**<br>♀ n = 6, ♂ n = 15	**MRC assessment for clinical ICUAW diagnosis**<br>♀ n = 6, ♂ n = 5

	**Observational Trial**			**Intervention Trial**					
				Intervention			Control		
	♂ n = 17	♀ n = 5	p-Value	♂ n = 15	♀ n = 6	p-Value	♂ n = 5	♀ n = 6	p-Value
ICUAW at first awakening (n,%)	13 (76.5)	3 (60.0)	0.124	14 (93.3)	6 (100)	0.517	3 (60.0)	6 (100)	0.087
ICUAW at discharge (n,%)	10 (58.8)	2 (40.0)	0.457	10 (66.7)	5 (83.3)	0.445	2 (40.0)	5 (83.3)	0.137
MRC at first awakening	3.3 (3.0/3.8)	4.4 (3.3/4.9)	0.090	3.4 (2.5/3.9)	2.2 (1.6/3.1)	0.138	3.3 (3.0/4.0)	3.0 (2.4/3.0)	0.224
MRC at ICU discharge	3.8 (3.1/4.4)	4.1 (3.6/4.9)	0.307	3.9 (3.0/4.0)	3.4 (2.3/3.9)	0.482	4.0 (3.8/4.1)	3.7 (3.1/3.9)	0.233

Results are reported as median with interquartile range or as absolute numbers with percentages. Mann-Whitney Test and Pearson Chi^2 Test. ICUAW: Intensive care unit acquired weakness, MRC Medical Research Council Score.

Table S2. Multivariable linear regression model for atrophy gene expression of Calpain-1 and Atrogin-1.

Model 1 (Unadjusted)					Model 2				
**Calpain-1**									
**Variable**	***n***	**B**	**95% CI B**	***p*-Value**	**Variables**	***n***	**B**	**95%CI B**	***p*-Value**
Sex	57	15.7	(-1.2 32.5)	0.410	Sex	57	13.1	(-4.2 30.4)	0.136
Constant		3.7	(-5.2 12.7)	0.069	Age	57	.3	(-0.2 0.7)	0.221
					Constant		-10.6	(-35.6 14.3)	0.396
n= 57 ICU patients, R=.243, R^2=.059 (adjusted R^2=.042), *p*=.069					*n*= 57 ICU patients, R=.292, R^2=.085 (adjusted R^2=.051), *p*=.091				
**Atrogin-1**									
**Variable**	***n***	**B**	**95% CI B**	***p*-Value**	**Variables**	***n***	**B**	**95% CI B**	***p*-Value**
Sex	58	31.8	(-2.7 66.2)	0.070	Sex	58	27.9	(-7.4 63.2)	0.120
Constant		6.4	(-12.3 25.1)	0.496	Age	58	.5	(-0.5 1.4)	0.306
					Constant		-18.5	(-70.1 33.2)	0.477
n= 58 ICU patients, R=.239, R^2=.057, (adjusted R^2=.040), *p*=.070					*n*= 58 ICU patients, R=.274, R^2=.075 (adjusted R^2=.042), *p*=.116				

Circulating triglycerides concentrations

Circulating triglyceride concentrations were retrospectively extracted from clinical routine laboratory files during the review process. Circulating triglyceride concentrations were available in n = 13 male and n = 5 female ICU patients. The triglyceride concentration measured at the closest time point to date of muscular glycerol determination was included. Median time gap was 6 (2/11) days. Circulating triglyceride concentrations taken during clinical routine did not correlate with glycerol measured in the skeletal muscle (Spearman-Rho n = 18, k = -0.190, R^2 = 0.046, p = 0.450) (Figure S2a). Median triglyceride level was 99.0 (85.0/170.0)mg/dl in male and 116.0 (103.0/130.0) mg/dl in female ICU patients (p = 0.805) (Figure S2b). Interpretation is limited as triglycerides measurements were not part of the study design. Consequently, circulating triglycerides have not been measured simultaneously to muscular glycerol and were not available in all patients.

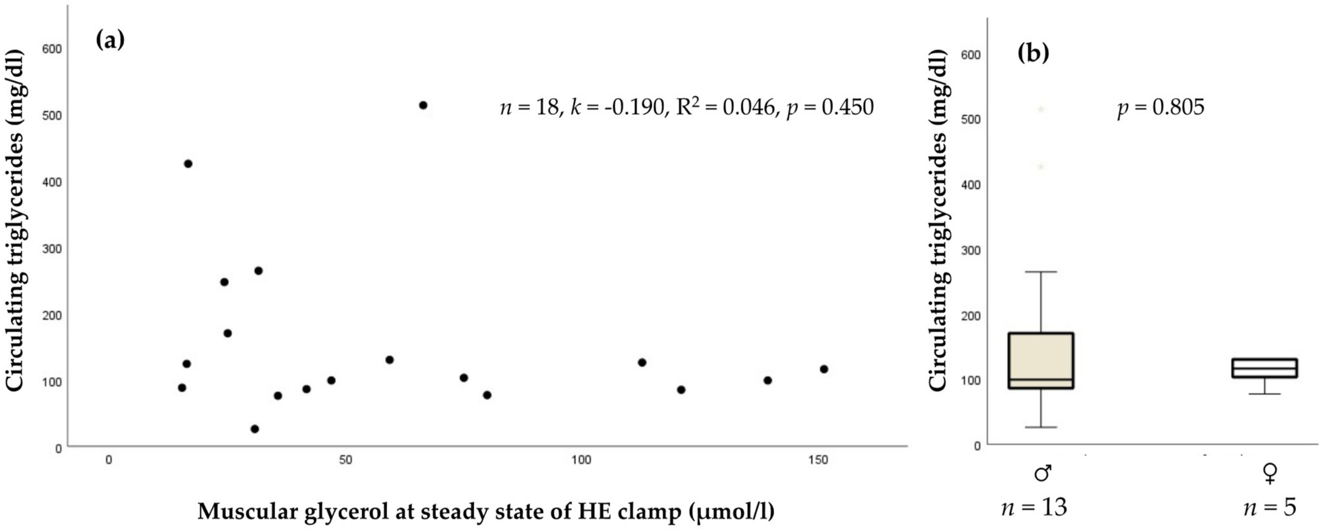

Figure S2. (**a**) Spearman´s correlation of circulating triglycerides and muscular glycerol by microdialysis in *M.vastus lateralis*. (**b**) Sex-specific circulating triglycerides. Mann-Whitney Test.
